# Chitosan-based hydrogels: From preparation to applications, a review

**DOI:** 10.1016/j.fochx.2023.101095

**Published:** 2023-12-27

**Authors:** Fandi Hong, Peng Qiu, Yufan Wang, Peirou Ren, Jiaxin Liu, Jun Zhao, Dongxia Gou

**Affiliations:** a College of Food Science and Engineering, Changchun University, Changchun 130022, China; b Jilin Province Product Quality Supervision and Inspection Institute, Changchun 130103, China

**Keywords:** Chitosan-based hydrogel, Crosslink, Bioactivity, Food applications

## Abstract

Chitosan, derived from the deacetylation of chitin, is an abundant natural biopolymer on earth. Chitosan and its derivatives have become promising biological materials because of their unique molecular structure and excellent biological activities. The reactive functional groups of chitosan such as the amino and hydroxyl groups play a crucial role in facilitating the synthesis of three-dimensional hydrogel. Chitosan-based hydrogels have been widely used in medical, pharmaceutical, and environmental fields for years. Nowadays, chitosan-based hydrogels have been found in a wide range of applications in the food industry such as food sensors, dye adsorbents and nutrient carriers. In this review, recently developed methods for the preparation of chitosan-based hydrogels were given, and the biological activities of chitosan-based hydrogels were systematically introduced. Additionally, the recent progress in food sensors, packaging, dye adsorbents, and nutrient carriers was discussed. Finally, the challenges and prospects for the future development of chitosan-based hydrogels were discussed.

## Highlights

• The preparation methods of chitosan-based hydrogels were intensively studied.
• The bioactivity and mechanism of chitosan-based hydrogels are described in detail.
• Frontier advances in the food application of chitosan hydrogels were provided.
• The development prospects of chitosan-based hydrogel in food packaging were given.

## 1 Introduction

Hydrogels are flexible and moist materials possessing a three-dimensional interwoven matrix (Liu et al., 2018, Liu et al., 2018), featuring a loose, interconnected porous structure that imparts remarkable water retention capabilities, defying dissolution. Moreover, owing to their exceptional elongation properties, hydrogels possess the unique potential to be molded into an assortment of shapes, granting them a versatile nature (Erol, Pantula, Liu, & Gracias, 2019). Hydrogels can be classified into two categories, distinguished by the origin of their constituent materials: natural hydrogels and synthetic hydrogels. Among these, natural polysaccharide hydrogels have garnered significant attention, driven by their impressive mechanical attributes, superior biocompatibility, facile degradability, and an array of other commendable traits that contribute to their rising prominence (Asadi, Alizadeh, Salehi, Khalandi, Davaran, & Akbarzadeh, 2018).

Chitosan (CS), derived through the deacetylation of chitin, emerges as a captivating deacetylated polysaccharide. At the molecular level, its intricately woven structure encompasses uniquely arranged -(1,4)-bonded d-glucosamine and *n*-acetyl-glucosamine units (Xu, Han, & Lin, 2017), marking them as the exclusive natural occurrences of such glucosamine units. Notably, CS stands as nature’s solitary cationic polysaccharide, distinguished by its extraordinary chemical constitution, which bestows upon it a repertoire of remarkable biological attributes including antimicrobial prowess, antioxidant activity, adhesive potential, and remarkable biodegradability.

**Fig. 1:**
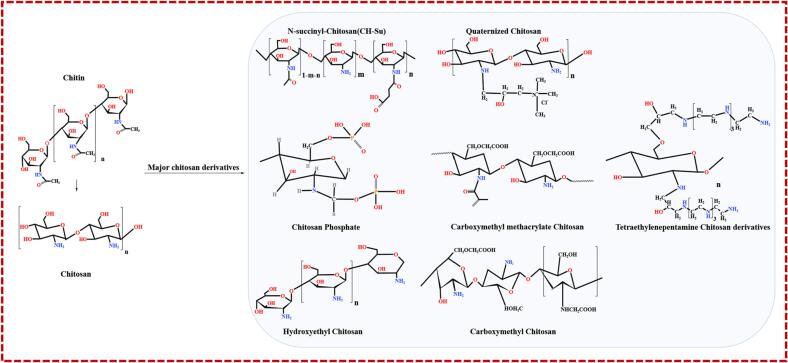
Structures of chitin, chitosan, and their major derivatives.

The remarkable antioxidant capabilities exhibited by chitosan stem from the presence of hydroxyl and amino groups (–NH_2_) intricately adorning its molecular chain. It is within this context that the amino groups effectively engage hydrogen atoms, evolving into ammonia ions (–NH_3_^+^), before forming steadfast macromolecules that diligently neutralize the pernicious effects of free radicals. Similarly, chitosan's unparalleled antibacterial characteristics (Guerini, Condro, & Perugini, 2022) result from electrostatic interactions between positively charged amino groups beautifully dispersed along its molecular chain and their negatively charged equivalents. This mesmerizing electrostatic attraction process instigates a cascade of damage to the cell membrane, unequivocally accounting for the observed antibacterial activity. While chitosan's manifold applications have witnessed widespread adoption, limitations persist in terms of its solubility, representing an ongoing challenge in the field. Chemical modification techniques have thus been employed to enhance the solubility of chitosan and enhance the cross-linking ability between polymer strands. Carboxymethyl chitosan (CMC), owing to its polyelectrolyte properties encompassing both positive and negative charges, remarkable water solubility, and significant chemical reactivity, emerges as a suitable option for constructing hydrogels in conjunction with other polymeric substances (Shariatinia, 2018, Shariatinia and Jalali, 2018). Hydroxyethylated chitosan is a chemical formed by substituting a hydroxyethyl group for the hydrogen atom in the chitosan molecule. The hydroxy ethylated chitosan molecule now has a hydroxyl group as the active center of the chemical process, conferring excellent water solubility and bioactive characteristics (Chalitangkoon, Ronte, & Monvisade, 2023). *N*-succinyl chitosan (CH-Su) was created by inserting a succinyl group at the *N*-position of the glucosamine unit, and it has greater cellular survival and biocompatibility than chitosan (Fig. 1).

The intricate network architecture of chitosan-based hydrogels offers a versatile platform for incorporating various bioactive compounds, thereby enabling a broad spectrum of applications in the realms of tissue engineering, drug delivery, food science, and beyond. With the ability to imbibe or contract in response to external stimuli, these hydrogels have demonstrated immense potential for a diverse array of fields (Zhao et al., 2023, Yang et al., 2023). Considering the growing prominence of green chemistry and the urgent need for environmental preservation, chitosan-based hydrogels have emerged as a promising alternative to conventional biodegradable materials. Their manufacturing simplicity, cost-effectiveness, and low toxicity render them highly advantageous. By harnessing the exceptional properties of natural polymers, chitosan-based hydrogels have the unique capability to retain the mechanical robustness characteristic of hydrogels. This amalgamation of attributes enhances their growth potential and contributes to their significant value across various applications (Lv et al., 2023, Zhang et al., 2023). With the rapid advancement of the food industry, chitosan hydrogels have garnered considerable attention due to their exceptional biological characteristics. These hydrogels hold great promise for a wide range of applications in food-related sectors, including food preservation, food processing, and food testing (Jinlong Li, Jia, & Yin, 2021). Consequently, this comprehensive study provides a review of recent research developments about the manufacturing techniques, bioactivities, and culinary applications of chitosan hydrogels, with a specific focus on the past five years. Additionally, this investigation aims to explore the variances and interconnections between different preparation processes and cross-linking methods employed for chitosan hydrogels. By doing so, this study strives to establish a robust theoretical framework for the future design and utilization of chitosan-based hydrogels in the food domain, supporting their continued advancement and progress.

## 2 Formation mechanism of chitosan-based hydrogels

Chitosan hydrogels, derived from chitosan and its derivatives, are natural polymer hydrogels formed through various physicochemical interactions such as hydrogen bonding and Schiff base reactions with other polymer compounds (Shariatinia and Jalali, 2018, Shariatinia, 2018). These hydrogels are categorized into different types based on their attachment mechanisms, including “reversible” or “physical” gels and “permanent” or “chemical” gels. Each classification signifies the nature of the gel and its stability.

### 2.1 Physical crosslinking

Physical cross-linking refers to the process of connecting chitosan polymer chains through non-chemical means or physical interactions, typically involving a combination of polymer and water components (J. Chen et al., 2019). These physically cross-linked chitosan hydrogels exhibit remarkable mechanical strength, reduced toxicity, and the ability to reverse their gelation process. In comparison to their chemically cross-linked counterparts, these physical hydrogels demonstrate superior biocompatibility and a lesser environmental impact (Table 1).

**Table 1:** Chitosan hydrogels prepared by physical methods.

Hydrogel name	Primary materials	Crosslinking method	Crosslinking agent	Application	characteristic	Citation
Cat-CH/Hydrogel	Sodium bicarbonate, chitosan、EDC	Physical cross-linking (ionic interactions)	Sodium bicarbonate	Injectable Adhesion Carriers	Fast gelation, high adhesion, good mechanical properties	(Guyot, Cerruti, & Lerouge, 2021)
OS Chitosan-based Hydrogel	Chitosan, tapioca starch, sodium periodate	physical crosslinking		Drug delivery vehicles	Good bacteriostatic properties with a slow-release effect, high solubility, and good cytocompatibility	(D. Sarmah, Rather, Sarkar, Mandal, Sankaranarayanan, & Karak, 2023)
SG/CS Hydrogel	Succinoglycan (SG), chitosan, 5-fluorouracil	Physical cross-linking (electrostatic interactions)		Ph Responds to Changes in Drug Delivery Systems	Good bacteriostatic properties and high cell viability; high biodegradability	(J. Kim, Kim, Jeong, Kim, Kim, & Jung, 2023)
Dual-crosslinked CMC-ALG hydrogels	Carboxymethyl chitosan, alginate, calcium chloride, EGF powder	Physical cross-linking (electrostatic interactions)		Clinical Wound Care Wound Aids	Promote cell proliferation, good blood compatibility, promote tissue regeneration	(Hu et al., 2018)
CCHs hydrogel	Carboxymethyl chitosan powder, allyl glycidyl ether (AGE), ammonium persulfate (APS), calcium chloride (CaCl_2_)	Physical cross-linking (hydrogen bonding interactions, ionic interactions)	Allyl glycidyl ether	As flexible sensors, wearable devices, and energy harvesting devices	High conductivity, sensitivity, dependability, and quick reaction time	(Yang Wang, Zhang, Gong, Zhao, Liu, & Zhang, 2022)
TEPA − COS hydrogel	Tetraacetylethylenediamine (TEPA), epichlorohydrin (ECH), low chitosan	Physical cross-linking (hydrogen bonding interactions)	TEPA		Good mechanical strength, strong chelating effect, and adsorption effect on hexavalent chromium.	(Mei, Zhang, Li, & Ou, 2019)
(Fe^3+^-PCS/CS-fHNTs NC hydrogel)	Acrylamide (AAm), acrylic acid (AAc), ammonium persulfate (APS), kaolin nanotubes (HNTs), hyperbranched polysiloxane (HSiv)）	Physical cross-linking (ionic interactions, hydrogen bonding interactions), chemical cross-linking	HSiv	For load-bearing structural materials	Excellent mechanical properties and significant self-resilience	(Li et al., 2020, Li and Zhuang, 2020)
CS-TPPhydrogel	Chitosan, tripolyphosphate (TPP), SQR22 dye, dimethyl sulfoxide (DMSO)	Physical cross-linking (electrostatic interactions)	TPP		Better preservation of photothermal effects, photostability, better cytocompatibility, and thermal validity	(Lim, Kai, & Lee, 2023)
CS/SP printing inks	Chitosan powder, two-component silicone elastomer (ACEO), sulfate (SP)	Physical cross-linking (electrostatic interactions)	SP	Composite chitosan/silk particle scaffolds	Good shape retention and cytocompatibility, high structural stability	(J. Zhang et al., 2018)
Chitosan hydrogel-modified cotton fabrics	Chitosan polymer, amylase, sodium bisulfite (Na_2_S_2_O_4_), monochloroacetic acid (CAA), sodium carbonate (Na_2_CO_3_), reactive dye (Supra rouge S-PX)	Physical cross-linking (electrostatic interactions)		A functional cotton	Significant antimicrobial activity, pH sensitivity, good mechanical properties	(Trad et al., 2018)

By evaluating the crosslink density and the stability of the internal network structure, physical hydrogels can be stratified into two categories: strong physical hydrogels and weak physical hydrogels. Strong physical hydrogels possess robust physical linkages within their polymer chains, which sustain their functionality even under certain circumstances. Through non-covalent interactions, including hydrogen bonding and van der Waals forces (Ou & Tian, 2021), these polymers form molecular network architectures in water, manifesting both solution and solid gel properties.

#### 2.1.1 Ionic interaction

Ionic interaction serves as a prominent approach for establishing physical cross-linking within chitosan, effectively influencing and augmenting the cross-linking between polymer chains. This results in the formation of a highly stable network structure, thereby enhancing the mechanical strength and stability of the chitosan hydrogel (Ravishankar & Dhamodharan, 2020). Moreover, ionic cross-linking provides a means to regulate the pore morphology and surface properties of the hydrogel, consequently impacting key parameters such as permeability. Exploiting the potential of ionic interactions allows for precise control over the morphologyical characteristics of the hydrogel, enabling energy storage switching mechanisms and expanding the possibilities in chitosan hydrogel compositions. For example, Mitsuhashi et al. (Mitsuhashi, Qi, Takahashi, Ohta, & Ito, 2019) successfully synthesized a chitosan-based hydrogel for the prevention of peritoneal adhesions by cross-linking *n*-succinyl chitosan (CH-Su) hydrogel with various multivalent metal ions (Fe^3+^, Al^3+^, and Ca_2_^+^). *N*-succinyl chitosan possesses abundant amino and hydroxyl groups, which can interact with metal ions. Upon introduction of positively charged metal ions, ionization occurs with the negatively charged amino and hydroxyl groups. Subsequently, the metal ions establish ionic pairs with the negatively charged functional groups in *N*-succinyl chitosan, leading to the cross-linking of polymer chains and the generation of a three-dimensional mesh structure. The results showed that the CH-Su/Al^3+^ hydrogel possessed remarkable mechanical properties, structural stability, a suitable degradation rate, and low cytotoxicity (the toxicity may be caused by the polycationic effect of chitosan, leading to the disruption of the lipid membrane structure). Based on FT-IR spectroscopy analysis, we found that CH-Su hydrogels have high binding capacity to Fe^3+^ and Al^3+^. This may be attributed to the fact that Fe^3+^ and Al^3+^ have higher charge densities, which are more favorable to react with the amide and carboxyl groups in CH-Su to form succinate-metal ion complexes.

#### 2.1.2 Hydrogen bonding interaction

Hydrogen bonding interactions play a pivotal role in the physical cross-linking of hydrogels, exerting significant influence over their structure, characteristics, and stability. These interactions arise when a positively charged hydrogen atom forms an electrostatic connection with a negatively charged acceptor atom, such as oxygen, nitrogen, or fluorine (Liu et al., 2018, Liu et al., 2018). Through hydrogen bonding, polymer chains are drawn together, resulting in the formation of non-conventional spatial arrangements within hydrogels. Consequently, hydrogels acquire unique shapes and mechanical properties, including elasticity and the ability to bear pressure-induced deformation. The breaking or formation of hydrogen bonding contacts assumes paramount importance when the surrounding environment changes, as they crucially impact the stability and shape of the hydrogel. Changes in temperature, pH, and ionic concentration, for example, can cause hydrogen bonds to form or break, affecting the characteristics and structure of the hydrogel (B. Tian & Liu, 2023).

Based on this theory, pH-sensitive, temperature-sensitive chitosan-based hydrogels are produced as smart hydrogels. By adjusting the reaction solution at appropriate acidic conditions, carboxymethyl chitosan and sodium alginate could form a network mainly through the hydrogen bonds, this hydrogel exhibited excellent pH sensitivity, pH reversibility and protein loading capacity (Jing, Huang, Du, Mo, Ma, & Wang, 2022). Due to the hydrogen bond between chitosan and polyacrylamide (PAM) network, short-chain chitosan can improve the mechanical properties of PAM hydrogels. Budianto E et al. (Budianto & Amalia, 2020) utilized chitosan, acetaldehyde, and NNMBA(N, *N*-dimethyl-bisacrylamide) to create pH-responsive chitosan hydrogels with interpenetrating polymer network architectures. The hydrogels had the highest degree of cross-linking and the most stable structure. Chitosan interaction with amino groups in acetone via the Schiff base reaction and hydrogen bonding in the hydrogel improved mechanical characteristics and mechanical strength.

Chitosan could also be modified by orotic acid through the carbonxyl group, and the CO-NH-CO group can form a complementary hydrogen bond with a suitable molecular scaffold. Using this method, a supramolecular hydrogel was fabricated by combining orotic acid-modified chitosan with 2,6-diaminopurine. The formation of the hydrogel is mainly driven by the intermolecular self-assembly of these components through hydrogen bonding. The resulting hydrogel exhibits dual responsiveness to temperature and pH and has great potential in the field of gastrointestinal drug release (Wang et al., 2023, Yang et al., 2023, Zhao et al., 2023).

#### 2.1.3 Hydrophobic interaction

The phenomenon of hydrophobic interactions, wherein hydrophobic molecules exhibit attractive forces, plays a significant role in shaping the assembly and properties of physically cross-linked hydrogels. The attractive forces are due to acid-base-free energy of cohesion among the water molecules. These interactions promote the formation of larger aggregates of molecules in solution, rather than existing as individual entities. Consequently, hydrophobic interactions enhance the intermolecular attraction among polymer chains, facilitating the development of physically cross-linked hydrogels and imparting enhanced stability and mechanical properties to the hydrogel network (Chang et al., 2018). Moreover, by modifying the microstructure within hydrogels, such as porosity and surface area, hydrophobic interactions directly influence the characteristics of the hydrogel. Notably, in conjunction with other key interactions like hydrogen bonding, hydrophobic interactions exhibit the ability to induce structural modifications and shape the properties of physically cross-linked hydrogels (He, Huang, Yang, Huang, & Wang, 2019). This multifaceted interplay of interactions expands the repertoire of possibilities for tailoring and engineering hydrogel materials with desired characteristics for a wide range of applications. For example, Han Y et al. (Han, Tan, Wang, Xu, & An, 2019) found that hydrophobic interactions also exist in synthetic polymers such as hydrophobically attached polymers (HAP) or hydrophobically attached polyelectrolytes (HAPE). To improve the hydrophobicity of these materials, several methods related to the molecular weight, the distribution sequence of hydrophobic groups, the type and content of hydrophobic groups, the type and content of ionic groups, and the relative positions between hydrophobic and ionic groups can be used. Enache A C et al. (Enache et al., 2022) prepared mycobacteria (NYSm) hydrogels using chitosan hydrogel as a loading carrier, with the amino groups on the chitosan chains and the hydrogen bonds between NYSm molecules physically crosslinked via hydrophobic and hydrogen bonding interactions. This hydrogel outperformed chemically manufactured hydrogels in terms of fungal inhibition, swelling, and drug release under various pH settings. Another typical method is to incorporate hydrophobic functional groups by modifying chitosan to enhance its interaction with hydrophobic substances. Huang L et al. (L. Huang et al., 2021) employed thiolate chitosan and maleic anhydride-treated chitosan to generate chitosan hydrogels. To lower the size of the hydrogel and boost its mechanical strength, it was primarily twice cross-linked utilizing click chemistry and sure physical forces (hydrophobic interactions, hydrogen bonding). The resultant hydrogels are non-toxic and have beneficial mechanical properties.

#### 2.1.4 Electrostatic interaction

Electrostatic interactions are one of the more common interactions in nature (Zhang et al., 2020, Zhang et al., 2020); when two charged objects are nearby, an electrostatic attraction or electrostatic repulsion is formed between them, affecting molecule aggregation and gel structure stability. Polyelectrolyte complexes (PECS) are generated by electrostatic interactions between cationic amino groups in chitosan and anionic groups in other polymers under pH conditions. Its magnitude is determined by variables such as the quantity and distribution of charge in the hydrogel's internal system, as well as the concentration and polarity of ions in the medium surrounding the hydrogel. A. Papa Giannopoulos et al. (Papagiannopoulos, Nikolakis, Pamvouxoglou, & Koutsopoulou, 2023) used the electrostatic association to create carrageenan/chitosan (Car/Chit) physical hydrogels. The electrostatic action created by the polysaccharide resulted in a decrease in the degree of swelling and an increase in the viscoelasticity of the hydrogels under acidic conditions, which is comparable with the results obtained with the addition of lap. Wang Y et al (Wang et al., 2023, Wang et al., 2023, Yang et al., 2023, Zheng et al., 2023) utilized chitosan with a deacetylation degree exceeding 90 % as the primary material. By introducing catechol groups onto the surface of mussel adhesion proteins through the electrostatic interaction method, they successfully harnessed the amino group on the chitosan chain to facilitate a joint reaction through electrostatic interactions with the sulfonic acid group of the thermo-sensitive monomer, *n*-isopropyl acrylamide. This novel approach resulted in the development of an injectable hydrogel that exhibits degradability, biocompatibility, and thermo-sensitive properties, while also demonstrating excellent adhesive properties. Chitosan-based hydrogel produced though electrostatic interactions can be employed not only in the creation of injectable gels, but also in the adsorption of food colors as positively charged cations. Wan X et al. (Wan et al., 2022) discovered the hydrogel production process via electrostatic interactions. With the addition of a cross-linking agent, a modest number of C=N bonds were formed between acrolein and chitosan (CS). This link causes a shift in hydrogen bonding within CS, with intramolecular hydrogen bonding decreasing and intermolecular hydrogen bonding increasing. The C=N bond is increased with the addition of PEI, and the hydrogen and C=N bonds cross-link to produce the mesoporous framework of the adsorbent A-PEI/CS. Under acidic circumstances, the protons of the amino group on the adsorbent increase, causing individual oxygen atom electrons to leave the orbitals of the dye benzene ring, resulting in an n- πelectronic force. This increases the adsorbent's positive charge density and attracts the antagonistic charge of the anionic dye (AB93), improving AB93 removal by the adsorbent.

### 2.2 Chemical crosslinking

The chemical gel is composed of a network connected by covalent bonds, with multiple amino and hydroxyl functional groups on the chitosan chain that may interact with the cross-linking agent to form a stable three-dimensional gel structure, and the formation of covalent bonds results in a gel structure that has more rigidity and stability (Bingren Tian, Hua, Tian, & Liu, 2020). Chemically cross-linked hydrogels (also known as true gels) are created by linking two polymer molecules with a covalent bond. Crosslinked molecules generate covalent units across polymer chains (Lu et al., 2022, Li et al., 2022, Lu et al., 2022), resulting in a hydrogel that is often permanent, irreversible, and stable. Polymer compounds easily cross-link due to the extremely reactive hydroxyl and amino groups on the chitosan chain. Furthermore, the active groups of derivatized chitosan can undergo new chemical cross-linking, giving it improved mechanical capabilities, structural stability, bacteriostatic qualities, and biocompatibility (Ardean et al., 2021). (Table 2).

**Table 2:** Chitosan hydrogels prepared using chemical cross-linking.

Hydrogel name	Primary materials	Crosslinking method	Crosslinking agent	Application	characteristic	Citation
API-CS-oxCS/oxHA	Chitosan, sodium periodate, hyaluronic acid	Chemical cross-linking (Schiff base reaction)	oxCS, oxHA	For wound care	Excellent self-healing ability, high stability, cytocompatibility, and bacteriostatic properties	(Tatarusanu et al., 2023)
Cn-Nm hydrogel	Chitosan, aldehyde-4-arm polyethylene glycol, amino-4-arm polyethylene glycol, potassium hydroxide, sodium chloride	Chemical cross-linking (Schiff base reaction)	4r-PEG-CHO	Antimicrobial wound aids for traumatic wounds	Better mechanical properties, swelling; very strong cytocompatibility and bacteriostatic properties	(Lu et al., 2022, Lu et al., 2022)
PVA/dextran/chitosan hydrogel	Glutaraldehyde, polyvinyl alcohol, chitosan, dextran	Chemical crosslinking	Glutaraldehyde	For healing of burns, bedsores, etc.	Gram-positive bacteria are inhibited. boost cell growth and has strong thermal and mechanical qualities	(Lin, Lo, Tseng, Liu, Shih, & Cheng, 2019)
Chitosan-based isothiocyanatotrimellitic anhydride cross-linked hydrogels	Br12 dye, chitosan, isothiocyanatotriphthalic anhydride	Chemical crosslinking	Isothiocyanatotrityltriphthalic anhydride	Treatment of BR − 12 Dyes Highly Effective Alternative Adsorbents	The extremely strong adsorption capacity for BR dyes	(Mohamed, Al-Harby, & Almarshed, 2021)
PC/CS （PCCS）hydrogels	Chitosan hydrochloride, carboxylated polyvinyl alcohol (PC), succinic anhydride, triethylamine (TEA)	Chemical cross-linking (amidation reaction)	Carboxy polyvinyl alcohol (PC)	Adhesive dressings for dynamic traumatic wounds	Good self-healing ability, strong adhesion, and antimicrobial ability; good cytocompatibility and hemostatic properties	(Liu et al., 2023, Liu et al., 2023)
BC-Ch Hydrogel	Genipin, chitosan, bacterial cellulose (BC)	Chemical crosslinking	Genipin	Drug carrier	Controlled drug release capability and swelling behavior	(Arikibe, Lata, Kuboyama, Ougizawa, & Rohindra, 2019)
Frozen-Thawed CMC-CS-CA hydrogels	Chitosan, carboxymethyl cellulose sodium salt,	Chemical and physical cross-linking (hydrogen bonding interactions, electrostatic interactions)	Citrate	A device for green storage of electrolytes	Improved ionic conductivity for better discharge performance	(Bósquez-Cáceres, Bejar, Álvarez-Contreras, & Tafur, 2023)
Hierarchical microCS hydrogel scaffold	Chitosan powder, LiOH, epichlorohydrin (ECH)	Chemical crosslinking	Epichlorohydrin	Stents mimicking the dura mater	High mechanical strength and degradation rate, promotes cell proliferation, good cytocompatibility	(J. Wang et al., 2021)
(CS-PEG-HA) hybridized hydrogels	Methacrylic anhydride and dibenzocyclotitani- peg4 - hydroxy succinimide ester (dbco - peg4 - nhss -ester), chitosan, 4-arm PEG-azide (2 KDa), 1-(3-dimethyl aminopropyl)-3-ethyl carbodiimide hydrochloride	Chemical crosslinking	Polyethylene glycol, methacrylic anhydride	Novel materials to accelerate wound healing in the body or on the skin	Excellent adhesion and hemostatic properties for faster skin healing	(Li et al., 2022, Li et al., 2022, Lu et al., 2022)

Chemical crosslinking of hydrogels enables the formation of stable gels with superior mechanical properties by covalently linking polymer chains. In contrast to physical hydrogels, these chemically crosslinked gels exhibit enhanced resistance to external environmental factors that may compromise the integrity of the gel structure (Eivazzadeh-Keihan et al., 2022). Consequently, the mechanical characteristics of hydrogels can be significantly improved.

#### 2.2.1 Schiff base reaction

In the majority of cases, the Schiff base reaction is conducted under acidic conditions to facilitate the condensation reaction between a ketone or alcohol and an amino group, resulting in the formation of amide molecules (Sang et al., 2021). A nucleophilic addition condensation reaction between the amine group of the chitosan and the aldehyde group of the catalyst occurs in the Schiff base reaction of modified chitosan with a catalyst to form a Schiff base-linked product (Lal, Arora, Kumar, Rani, Sharma, & Kumar, 2018). Notably, the Schiff base reaction holds great potential for generating three-dimensional network structures through cross-chain reactions that establish cross-linking connections between polymer molecules. The resulting cross-linked hydrogels exhibit good mechanical strength and structural stability. Furthermore, the Schiff base reaction offers the potential to tailor the degree of cross-linking and structural characteristics, enabling precise control over the rate and mechanism of drug release.

**Fig. 2:**
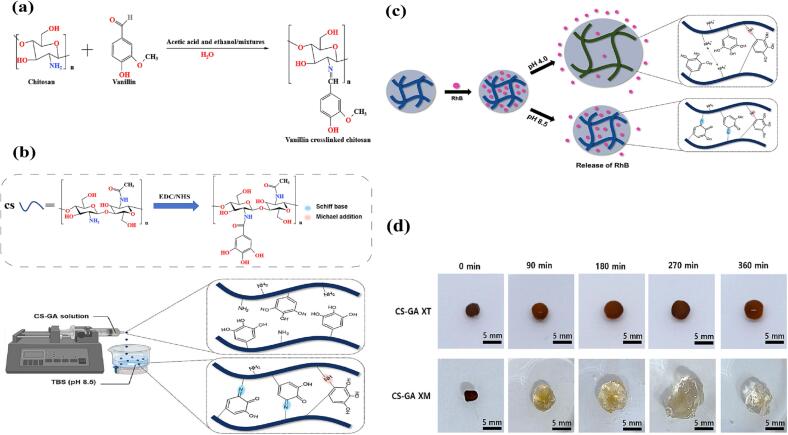
(a) Schiff base reaction between vanillin and CS (b) Synthesis of CS-GA conjugates and preparation of CS-GA gel beads (c) Schematic representation of swelling and drug release of CS-GA beads upon pH change (d) Prepared CS-GAXT and CS-GAXM beads (Reproduced with permission from ref (Park et al., 2023)).

Park G R et al. (Park, Gwak, Choi, & Park, 2023) effectively synthesized CS-GA conjugate by grafting gallic acid (GA), a natural polyphenolic molecule, onto the backbone of chitosan (CS) using 1-Ethyl-3-(3-dimethyl aminopropyl) (EDC)and *N*-Hydroxy succinimide(NHS). Following that, CS-GA hydrogel beads were created by dropping a sol of this conjugate into a tri-borate buffer solution (TBS). During the conjugate synthesis, EDC activated the carboxyl group of GA and interacted with NHS to generate a stable derivative, which then reacted with the amino group of CS to form an amide bond, resulting in the creation of CS-GA conjugates. The Schiff base reaction caused a considerable variation in dissolving rates between the CS-GA hydrogel beads at pH 4 (CS-GAXM) and pH 8.5 (CS-GAXT) (Fig. 2). Chitosan-based hydrogels reacted with Schiff base have good mechanical strength bioactivity in addition to controlled drug delivery. Dziadek M et al. (Dziadek et al., 2022) generated CS-PC/A2/RA hydrogels by cross-linking them with 2,3,4-trihydroxybenzaldehyde (THBA) and then modifying them with pectin (PC), biologically active glass (BG), and rosemarinic acid (RA). The three hydroxyl groups in THBA offered extra binding sites with other components under acidic circumstances, while the group of aldehydes produced imine bonds with the amino group on the chitosan chain. Electrochemical interactions between PC and CS also resulted in the formation of electrolyte complexes. The positively charged hydrogen atoms of CS hydrogen bonded with the negatively charged oxygen atoms of RA, while the calcium ions in BG electrostatically interacted with the carbonyl bonds in pectin, resulting in multiple synergistic interactions. The Schiff base’s reaction, on the other hand, is more intense than ionic contacts and hydrogen bonds. The generated hydrogels have great mechanical qualities, and strong antioxidant properties, are cytotoxic, and can even suppress cancer cell development.

#### 2.2.2 Diels-Alder reaction

The Diels-Alder reaction holds a crucial role in synthesizing a wide range of natural products, medicinal compounds, and complex chemicals. Typically, this reaction involves the asymmetric combination of a non-conjugated diene and an allylene, resulting in the formation of a novel six-membered ring structure (T. Wang, Naredla, Thompson, & Hoye, 2016). Notably, the reaction can proceed through either initial or final additions to the conjugated diene component. Diels-Alder reaction-modified chitosan hydrogels can have a wide range of features, including injectability, self-healing, and high mechanical qualities (O. Guaresti, Garcia-Astrain, Aguirresarobe, Eceiza, & Gabilondo, 2018). This is owing to changes in the chemical structure of the chitosan-based hydrogels caused by newly inserted groups or alterations to the existing groups. These modified chitosan-based hydrogels have a wide variety of uses as catalyst carriers, which may considerably boost reaction rate and improve reaction product purity (Zhang et al., 2020, Zhang et al., 2020).

Li D et al. (C. H. Chen et al., 2021) created pectin-chitosan hybrid hydrogels. Furfural was mainly used to modify pectin to obtain conjugated diene fractions and to graft 6-maleimidohexanoic acid onto chitosan to produce protodiol fractions. Hybridized hydrogels were prepared by combining furfural-modified pectin (PF) with maleimide-modified chitosan (CA) using the Diels-Alder process. The results showed that the hydrogels were able to carry 500 g without damage. The hydrogels have excellent self-healing properties, which may be attributed to the rapid physical action that occurs when the hydrogels are damaged, thus accelerating the recovery process of the hydrogels. In addition, the hydrogels have good pH and temperature responsiveness. This may be related to the ionization of pectin and chitosan under different pH conditions since both pectin and chitosan are amphoteric compounds under the Bronsted-Lowry acid-base theory. In an acidic environment, –COOH in pectin molecules and –NH_2_ in chitosan molecules exist as –COOH_2_^+^ and –NH_3_^+^. When the pH increases above 7, they transform to –COO^-^ and –NH_2_.

#### 2.2.3 Michael addition reaction

The Michael addition reaction, also known as the Michael reaction, is a conjugate addition reaction involving the nucleophilic reagent and an α, β-unsaturated compound that possesses one or more carbon–carbon double bonds with functional groups attached (G. Huang & Li, 2017). This versatile reaction finds extensive applications in the synthesis of natural products, pharmaceutical compounds, and materials. Its broad utility stems from its ability to introduce diverse functional groups onto conjugated systems, facilitating the creation of complex molecular architectures and enabling the development of novel chemical structures with potential applications in diverse scientific disciplines. Guar Esti et al. (Olatz Guaresti, Basasoro, González, Eceiza, & Gabilondo, 2019) effectively produced water-soluble chitosan derivatives by sulfuryl ting the parent polymers using thiolactic acid. Then, utilizing a sulfhydryl-Michael addition process, a pH-sensitive chitosan-based hydrogel was created by covalently cross-linking sulfhydryl-modified chitosan (CsSH) with water-soluble bis maleimide (BMI). The hydrogels demonstrated outstanding mechanical characteristics and structural stability, as well as good in vivo degradability and thermal stability. Increasing the quantity of water-soluble bismaleimide in the hydrogel dramatically improved the storage modulus value of the hydrogel and hastened its disintegration process.

#### 2.2.4 Thiol-ene reaction

The thiol-ene reaction, also known as the thiol-ene click reaction, involves the substitution of a hydrogen atom on the sulfur atom of a thiol group with a carbon atom on the carbon–carbon double bond of an olefin, resulting in the formation of a new carbon–sulfur single bond. This transformative reaction is particularly valuable for the modification of polymers, leading to the generation of novel functional materials and bioactive compounds (Summonte, Racaniello, Lopedota, Denora, & Bernkop-Schnurch, 2021). By introducing sulfur-containing groups into polymer structures, the thiol-ene reaction opens new avenues for tailoring and optimizing the properties and functionality of polymers, enabling advancements in diverse fields such as materials science and biotechnology. Li R et al (R. Li et al., 2018) used the mercaptoene reaction to effectively produce liposome/chitosan hydrogels of thioglycolate chitosan-coated liposomes and chitosan-maleate-coated liposomes. The results indicated that the produced hydrogels had a porous structure, and samples with varying *n*-SH / *n*-C=C ratios had variable gelation periods and swelling rates. The NMR spectra revealed that following modification with methacrylic acid, the protons of the CH = CH group displayed novel chemical shifts compared to pure chitosan, showing that MA was successfully modified. The hydrogels improved in mechanical strength, surface roughness, and biocompatibility with the inclusion of liposomes, and the crystal structure of the hydrogels grew smaller, more compact, and more stable. In contrast with pure chitosan hydrogels, chitosan-based hydrogels prepared through physical or chemical cross-linking improved the mechanical strength, application range, and physical and chemical characteristics of the gels themselves, enhancing the functional properties and practical application value of chitosan-based hydrogels.

#### 2.2.5 Nucleophilic ring opening reaction

Nucleophilic addition reactions involve the reaction between nucleophilic reagents, such as alcohols, amines, and thiols, and regions of high electron density in the reactants. This leads to the disruption of the ring structure and the formation of new chemical bonds, resulting in the creation of new compounds or the expansion of the ring structure. The reaction primarily occurs through the nucleophilic attack of ternary heteroatoms (such as oxygen, nitrogen, sulfur) within the molecule, overcoming cyclic strain and releasing internal energy to form ions like cyclic sulfur ions, cyclic sulfates, and epoxidized heterocyclic propane compounds (Kaur, Saxena, & Rishi, 2021). These reactions exhibit good selectivity and can be conducted under mild conditions, either in protonated aqueous solutions or in the absence of solvents. Wang C et al. (C. Wang, Yu, Chen, Zhang, Wang, & Liu, 2019) successfully prepared 2-N,6-O-SCS by modifying chitosan with formamide, formic and chlorosulfonic acids, and N, *N*-dimethylformamide, then assembled on gelatin sponges to create 2-N,6-O-SCS-encapsulated scaffolds and tested their ability to capture VEGF growth factor as a cytokine reservoir. The chlorosulfate group in chlorosulfonic acid was protonated and created a positively charged intermediate during the sulfation modification of chitosan. The hydroxyl group in chitosan works as a nucleophilic site to attack the positively charged sulfate group, causing the oxygen-sulfur connection to be broken and an ester bond to be formed between the chitosan molecule and the sulfate group. The hydroxyl group of the nucleophilic site is therefore replaced by the sulfate group, resulting in sulfated chitosan.The findings demonstrated that sulfated chitosan-coated scaffolds could successfully collect VEGF growth factors in blood arteries in situ. Furthermore, the scaffold has the potential to stimulate angiogenesis without the need of foreign cells, which it does by restricting intravascular dermal growth factors in certain locations.

## 3 Bioactivity of chitosan-based hydrogels

Because of its unique qualities, such as biodegradability, low toxicity, and renewability, chitosan has been chosen as an alternative ingredient for the manufacture of hydrogels in many circumstances. Thus, in some circumstances, bioactivity may be advantageous. Chitosan hydrogels in food engineering are primarily constituted of natural polymers with strong antioxidant characteristics, considerable bacteriostatic capabilities, and acceptable cytotoxicity (Klosinski et al., 2022).

### 3.1 Antioxidant properties

Antioxidants play a crucial role in food processing and preservation by extending the shelf life of food products while maintaining their flavor and nutritional value. However, the use of synthetic chemical antioxidants raises concerns regarding potential adverse effects on human health. Consequently, there is a growing demand among consumers for natural and health-promoting antioxidant alternatives. In this context, chitosan, a natural polysaccharide, emerges as a highly desirable option owing to its inherent antioxidant properties (Muthu, Gopal, Chun, Devadoss, Hasan, & Sivanesan, 2021). By harnessing the antioxidant capabilities of chitosan, food manufacturers can meet the increasing consumer demand for natural and safer antioxidant solutions, thereby ensuring the production of healthier and more sustainable food products.

For example, Hashim A F et al. (Hashim et al., 2019) utilized chitosan and alginate as raw ingredients to create a microbead loaded with Omega-3 s (fish oil, natural hemp oil) and natural antioxidants (curcumin). The microbeads' antioxidant activities were evaluated using the DPPH assay and the peroxide value. The antioxidant capabilities and peroxide value of the samples were dramatically increased with the addition of curcumin due to polyelectrolyte complexation between cationic chitosan and anionic alginate. Complex formation between chitosan and polyethylene glycol effectively delayed the turmeric release process, safeguarding fish oil's antioxidant activity during oxidation. LI J et al. (J. Li, Zhao, Zhu, & Zhang, 2023) produced chitosan gelatin frozen gelatin hydrogels using the chemical tannic acid as a cross-linking agent and tested their antioxidant capabilities by measuring peroxide and thiobarbituric acid (TBA) levels. The results revealed that chitosan and gelatin increased with time at room temperature, while the antioxidant values of hydrogels rose significantly slower than their separate samples. This is due to the hydrogels' three-dimensional network structure as well as the tannins' numerous hydroxyl groups, which improve the hydrogels' antioxidant capacity. The hydrogels created can be employed as an effective chain-breaking antioxidant.

### 3.2 Anti-bacterial properties

Food safety has always been a significant concern within the food industry, as bacterial contamination can lead to food spoilage and illness. Therefore, the identification and utilization of natural and potent bacteriostatic agents are critical in addressing this issue (Zhang et al., 2023, Zhang et al., 2023). Chitosan, a naturally occurring polysaccharide derived from marine sources, holds remarkable potential in this regard. The positively charged chitosan molecules possess the ability to interact with negatively charged microbial cell membranes, thereby disrupting their integrity and causing the release of bacterial intracellular components. This mechanism exhibits profound bactericidal and bacteriostatic properties, effectively inhibiting bacterial growth and proliferation (Li and Zhuang, 2020, Li et al., 2020). The utilization of chitosan hydrogels as food bacteriostatic agents holds immense potential and wide-ranging applications. By meticulously evaluating the bacteriostatic and anti-biofilm capabilities of chitosan hydrogels, researchers can determine the optimal dosage and application methods (M. Kim, Shin, Sung, & Kadam, 2022). This ensures effective prevention of bacterial growth in food and eradication of biofilm formation, thereby preserving food quality and enhancing safety standards. Concurrently, delving into the antibacterial characteristics of chitosan hydrogels lays the groundwork for the development of novel antibacterial agents, bolstering the overall health of the food sector.

For example, Hao PY et al. (Hao et al., 2023) generated a turmeric cyclodextrin-grafted chitosan hydrogel by attaching curcumin to chitosan. The antimicrobial activity of the hydrogel against Staphylococcus aureus and Escherichia coli was found by antibacterial assay, which could be attributed to the electrostatic interactions between –NH_3_^+^ in the hydrogel and the negatively charged bacterial cells, thus inhibiting the growth and even death of the microorganisms. Since Gram-positive bacteria contain many peptides in their cell walls, whereas Gram-negative bacteria contain lipids, this may be the reason for the better inhibitory effect of the hydrogel against S. aureus compared to E. coli. Wang M et al. (Wang et al., 2023, Wang et al., 2023) created chitosan nanohydrogels with tanshinone to improve antibacterial and anti-biofilm activity against Streptococcus pyogenes. Because the remaining amino group of chitosan can respond as a responsive portion upon protonation, the hydrogel has a superior release ability under acidic circumstances. The hydrogel penetrates cell membranes and suppresses biofilm formation directly. When in a biofilm-forming environment, it also produces tanshinone to exert its antimicrobial activity and limit bacteria development. After 48 h, the biofilm formation rate was investigated, and the findings revealed that the usage of the hydrogel could suppress biofilm development for an extended length of time, obtaining an inhibition rate of 28.81 %.

### 3.3 Biocompatibility

Chitosan hydrogel, a biocompatible natural polysaccharide polymer, is widely employed as a food additive in the food industry. This usage aligns with the growing demand for safe, non-toxic, and biocompatible food additives (Stefanowska, Wozniak, Dobrucka, & Ratajczak, 2023). Assessing the biocompatibility of chitosan hydrogel is crucial to understanding its impact on human health during daily use. Moreover, these assessments serve as a reference point for the development of new food additives. Previous research has usually employed cytotoxicity to test the biocompatibility of produced hydrogels, with the MTT technique being a frequently used approach (C. H. Chen et al., 2021). The MTT technique is frequently used to analyze the effect of chitosan hydrogels generated by physical cross-linking procedures on cell metabolism (Rodriguez-Rodriguez et al., 2020). Electrostatic spinning was used by Liu Y et al. (Y. Liu et al., 2022) to create antimicrobial hydrogels for food packaging from chitosan, gelatin, and 3-phenyl lactic acid. The researchers looked at the antibacterial characteristics and biocompatibility of hydrogels in frozen chicken flesh. The toxicity of the created hydrogels on Caco-2 and SW480 cells was assessed using the MTT test, and the findings revealed that the cell survival rate of the generated hydrogels was higher than 90 % following co-culture. This implies that the hydrogel comprising 3-phenyl lactic acid, chitosan, and gelatin is a safe food packaging material with great biocompatibility. In addition, the study investigated the hydrogels' antibacterial activity, particularly against frozen chicken flesh. Using sodium bicarbonate as a cross-linking agent, Fletes-Vargas G et al. (Fletes-Vargas et al., 2023) effectively synthesized chitosan hydrogels with varying concentrations and molecular weights. The MTT test was used to analyze the indirect cytotoxicity response of chitosan hydrogels with HT-29 cells as target cells, and the findings revealed that after 24 h of incubation at 37 °C, the various hydrogel extracts produced demonstrated good cellular metabolic activity. The results demonstrated that chitosan hydrogels of various concentrations and molecular weights were cytotoxic-free. It suggests that chitosan hydrogels are non-cytotoxic, have high cytocompatibility, and promote cell growth. Selenium nanoparticles are nanoscale selenium materials with antibacterial, anticancer, and antioxidant properties. The concentration-dependent toxicity of selenium nanoparticles on cells, however, remains debatable. By incorporating selenium nanoparticles (SeNPs) into CS/GP hydrogels, Wu L et al. (L. Wu et al., 2021) created a new hydrogel. The experimental results revealed that the insertion of SeNPs increased the mechanical strength and mechanical characteristics of the hydrogel as well as expedited its molding process. Meanwhile, SeNPs improved the hydrogel's antioxidant characteristics. However, because excessive concentrations of SeNPs exhibited harmful effects, the appropriate concentration of SeNPs must be found to provide excellent cytocompatibility.

## 4 Chitosan based hydrogels in food applications

Chitosan-based hydrogels exhibit exceptional antibacterial and antioxidant properties, along with excellent biodegradability, rendering them highly suitable for incorporation into smart food packaging systems (Flórez, Guerra-Rodríguez, Cazón, & Vázquez, 2022). These hydrogels have garnered significant attention and application across diverse sectors due to their remarkable attributes, including heightened sensitivity, selectivity, rapid response time, and compact size (S. Wu, Wu, Zhang, Feng, & Wu, 2023). Specifically, chitosan-based hydrogels have found extensive utility in biosensor development, food preservation techniques, adsorption of food colors, and targeted nutrition delivery, as highlighted (Table 3).

**Table 3:** Chitosan-based hydrogels in food applications.

Hydrogel Name	Primary materials	Main physical and chemical standards	Application purpose	Citation
Chitosan-Sea Urchin Spiny Composite Membrane	Chitosan (high viscosity), sea urchin spiny powder (SUSP)	Infrared spectroscopy, thermogravimetric analysis, contact angle, oxidation resistance	As an alternative to producing hydrophobic and highly stable films	(Hamil et al., 2020)
CS/PVA-green tea extract/HβCD-IC	Chitosan (medium molecular weight), polyvinyl alcohol, tetraethyl orthosilicate (TEOS), 2,2 ' -diphenyl-1-pyridine hydrazide (DPPH-), 2,2-azinoquinoline (3-ethylbenzothiazoline-6-sulfonic acid) (ABTS^+^)	Green tea polyphenol composition analysis, infrared and XRD ray spectroscopy, mechanical properties, swelling ratio, antioxidant, in vitro release capacity	Used as a potential material for nutraceuticals and cosmetics	(Chuysinuan, Chunshom, Kotcharat, Thanyacharoen, Techasakul, & Ummartyotin, 2021)
Chitosan-based hydrogel	Chitosan, potassium persulfate, acrylamide	Infrared spectroscopy, dissolution rate, BSA release rate, stationary adsorption isotherms	Substances for process control and food supplement capsules for cholesterol and blood pressure control	(Varnier, Vieira, Wolf, Belfiore, Tambourgi, & Paulino, 2018)
CS-based composite hydrogels	Chitosan, Glutaraldehyde (GA), rhodamine B (RhB), methylene blue (MB)	Dissolution rate, degree of dye removal	Adsorption of dyes as self-assembled composite hydrogel materials in wastewater	(R. Wang et al., 2020)
CH	2,2,6,6-tetramethyl-1-piperidinyl oxy (TEMPO), citric acid (CA), N-(3-dimethyl aminopropyl)-N'-ethyl carbodiimide hydrochloride (EDC), *N*-hydroxy succinimide (NHS), chitosan (CS), glutaraldehyde (GD) tetracycline (TC)	Pair of TC adsorption experiments, basic characterization	As an adsorbent for the detection and removal of antibiotics from water	(Luo et al., 2022)
CS-FA-DN	CS (degree of deacetylation ≥ 95 %)、 FA (≥99 %)、 laccase、lysozyme（BR）	Loading efficiency and capacity, in vitro simulated digestion, photothermal degradation capacity	Expanding the use of ferulic acid in food applications while acting as an inhibitor of glycosylation end-products	(Zheng et al., 2023, Wang et al., 2023)
pH-responding hydrogel	Chitosan, Sericin、 acrylic acid hydrochloric acid、PETase	The sol–gel content analysis, FTIR, solubility, PETase release mechanism, SDS-PAGE analysis	Transportation of PETase to the colon to promote microplastic degradation in the gastrointestinal tract	(Ullah, Lee, Kim, Kwon, & Lim, 2023)

Hydrogel biosensors can be categorized into two distinct groups based on their mode of operation. The first group comprises stimulus-responsive hydrogels that do not possess intrinsic receptors. Instead, these hydrogels exhibit volume changes or phase transitions in response to external stimuli, including pH variations, temperature fluctuations, the presence of salt ions, electric fields, magnetic fields, and other similar factors (J. Yang et al., 2021). In contrast, the second group of hydrogel biosensors, known as passive hydrogels, incorporates receptors or reactive substances like ions, nanoparticles (NPs), bioactive compounds, or cells. These components enable passive hydrogels to detect and analyze biochemical and biological interactions with high precision (X. Liu, Liu, Lin, & Zhao, 2020).

### 4.1 As a food sensor

Biosensors play a vital role in the food supply chain as they enable the monitoring of food freshness and perishability. This capability allows for effective management of food safety and quality by detecting specific compounds and assessing their presence and concentration levels. In this context, hydrogel-based indicators hold significant promise due to their high hydrophilicity and water content. These characteristics are particularly advantageous as they enhance the responsiveness of the indicators (Junjun Zhang et al., 2021). It is worth noting that the water content in the hydrogel greatly influences the sensitivity and efficacy of certain indicators that rely on color shifts. By strategically placing pH sensors in proximity to food items, microclimate parameters such as pH, temperature, and humidity can be accurately measured. Such data collection is crucial for ensuring food safety and maintaining optimal freshness levels (Rungsima, Boonyan, Klorvan, & Kusuktham, 2020).

#### 4.1.1 pH-responsive chitosan hydrogel sensors

The pH value of a food product serves as a crucial indicator of its acidity or alkalinity, providing valuable insights into changes that occur within a specific environment (Price et al., 2020). This attribute directly correlates with important factors such as shelf life, product quality, and overall freshness. In the food packaging process, pH monitoring plays a vital role in ensuring that freshness criteria are consistently met. Given the significance of pH as an indicator of food safety and quality, the utilization of pH-sensitive responsive hydrogels has emerged as a highly effective approach within the realm of food packaging materials (Nakazawa, Wada, Fukushima, Tanaka, Kono, & Okazaki, 2020). These pH-responsive hydrogels exhibit exceptional capabilities in promptly and efficiently releasing bioactive compounds compared to conventional hydrogels. Chitosan can be pH sensitive in acidic (pH = 6.2) food packaging. Chitosan swelling can be enhanced due to its hydrophilic nature and capacity to function as a mediator to help in the transit of H^+^ or OH^–^ ions as well as bind and protonate with –NH_2_ molecules (Cui, Xi, Wang, Tan, Li, & Li, 2023).

Athauda T et al. (Athauda, Banerjee, & Karmakar, 2020) developed chitosan hydrogel-attached chipless radio frequency identification (RFID) resonators, for example, to provide the hydrogel with certain electromagnetic characteristics at ultra-wideband frequencies. The sensor is designed to operate in the pH range of 4 to 10, allowing it to be used for both acid and base detection. Under acidic circumstances, hydrogels expand and shrink in amplitude under alkaline conditions. Chitosan's hydrophilicity works as a mediator for the transfer of H^+^ or OH– (acidic/alkaline) and its binding and protonation with NH_2_ molecules under acidic circumstances (Ostrowska-Czubenko, Gierszewska, & Pieróg, 2015). Furthermore, the capacity of chitosan-based hydrogels to release active compounds under various pH settings is a significant application. Li H et al. (Li et al., 2022, Li et al., 2022) used chlorella as a filler to create chitosan/chlorella hydrogel beads by physical cross-linking. The gel beads showed the greatest degree of swelling at pH 6–8, according to research on their swelling at different pH levels and their propensity to emit corrosive acids. This might be owing to the gel's three-dimensional network structure and Chlorella's water absorption, while electrostatic interactions between the NH_3_^+^ held by chitosan itself attract the gel's crosslinked network, resulting in increased swelling. Experiments on pH-responsive controlled release demonstrated that the hydrogel beads could adapt to changes in the pH of the external solution and had higher water absorption characteristics, resulting in more effective active ingredient release.

#### 4.1.2 Temperature-sensitive chitosan hydrogel sensors

Temperature is a critical parameter across various scientific disciplines and industrial processes. The remarkable temperature-responsive properties of hydrogels enable their application in real-time temperature monitoring of both environmental conditions and liquid systems. Additionally, hydrogels serve as effective tools for controlling temperature during reactions or processing, facilitating precise control and enhancing product performance optimization (Qu et al., 2023). Moreover, hydrogels can serve as temperature indicators, offering valuable insights through observations of shape changes. This capability finds relevance in areas such as medication release and tissue engineering repair, where temperature-responsive hydrogels can be utilized to enable targeted and controlled delivery of therapeutics or facilitate tissue regeneration (C.-X. Zhao et al., 2022). The utilization of hydrogels as temperature-responsive indicators presents promising avenues for research and innovation in a wide range of fields, including biomedical engineering, drug development, and advanced materials science. Bi S et al. (Bi et al., 2020) synthesized chitosan solution (HBC) in a homogenous reaction system containing KOH and urea using epoxy butane. Chitosan nanohydrogels were formed by hydroxy butyl modification of chitosan solutions at various temperatures. In deionized water, the produced chitosan derivatives may accomplish amphiphilic self-assembly, and this transformation process is stable and reversible concerning ambient temperature variations. The hydrogels have superior biocompatibility and degradability when compared to standard hydrogels, as well as reversible self-assembly and deconstruction under temperature change and stable and adjustable thermochromic characteristics. The experimental results revealed that as the temperature rises, the color of the hydrogel progressively changed from clear to opaque, indicating that the high temperature increased the hydrophobicity within the nano hydrogel. The hydrogel gradually dissolved from a homogeneous and fully spherical shape into smaller particles as the temperature lowered, demonstrating that the hydrogel structure altered with temperature.

#### 4.1.3 Humidity chitosan hydrogel sensor

Hydrogel is a highly absorbent substance, and the humidity in the surrounding environment influences its production process, water absorption, capacity to release water, storage and structural stability, and response pace. Hydrogels' performance and applicability may be maximized by adequately managing their humidity (Ahmed, 2015), allowing them to play the optimal function in various applications. Shao P et al. (Shao, Wu, Chen, Sun, & Gao, 2020) created an edible membrane with a bilayer structure capable of unidirectional humidity management, with a barrier layer on the outside and a moisturizing layer on the inside, using hydrophobic ethyl cellulose and hydrophilic carboxymethyl chitosan hydrogel as ingredients. The exterior layer was generated by solvent casting with ethyl cellulose (EC) film, while the inside side was built of carboxymethyl chitosan and genipin, a non-toxic and highly biocompatible natural cross-linking agent. The prepared cross-linked films were proven to have good load-bearing capability and flexibility. The inner layer interaction of chitosan and genipin resulted in a drop in amino content, a decrease in water solubility, and an increase in the mechanical characteristics of the cross-linked membrane. By regulating the humidity, the prepared membrane was able to maintain the quality of shiitake mushrooms, inhibit mold growth, reduce oxidative damage during storage, and promote the increase of phenylalanine deaminase (PAL) activity, thereby stimulating the biosynthesis of phenolics and anthocyanins.

### 4.2 As a food packaging material

Food preservation is essential for people's health, and food packaging is essential for maintaining long-term freshness. Effective food packaging may separate food from the outside world, preventing bacterial and oxygen contamination, as well as offer a physical barrier to protect food from external environmental conditions (T. Huang, Qian, Wei, & Zhou, 2019). Traditional plastic polymer packaging has good mechanical strength, but because many of its monomers are polymeric organics, it cannot be completely degraded in a short period, has a low release rate of biologically active substances, and has a low recycling rate, all of which can easily cause environmental pollution. As a result of the foregoing, chitosan hydrogels have emerged as a viable alternative to plastic packaging. Through stimulation reactions (temperature, pH responsiveness, magnetic responsiveness, etc.), chitosan hydrogel releases encapsulated bioactive substances, improving the bioavailability, stability, and degradation rate of the active substances and the material itself, and achieving the goal of reducing environmental pollution (Y. Zhang, Dong, Liu, Wu, Pan, & Liu, 2022).

Food packaging materials include antimicrobial and antioxidant components that can be used as chemical-free preservatives, thereby increasing the shelf life of food items. Chitosan-based products, such as chitosan hydrogel films, have been widely used in this application. (Pérez-Córdoba, Norton, Batchelor, Gkatzionis, Spyropoulos, & Sobral, 2018). Natural polymer hydrogels can be used to absorb water or exudates inside the package due to water loss caused by the transpiration of fruits and vegetables and physicochemical changes in the packaged food, and hydrogels can be widely used for the preservation of fruits, vegetables, and meats by controlling temperature changes inside the package. Maroufi LY et al. (Maroufi, Tabibiazar, Ghorbani, & Jahanban-Esfahlan, 2021) established hydrogels of double aldehyde guar gum (DAGG) and pomegranate peel extract (PEE) following chitosan (CS) modification utilizing physical crosslinking Schiff base reaction, to employ them for food preservation due to their water absorption capacity. Gel characterization studies demonstrated that the hydrogel had a loose and porous structure, as well as improved solubility, mechanical strength, antioxidant activity, and bacteriostatic activity when compared to traditional chitosan hydrogels. As a result, it may be utilized as an absorbent matting material to absorb water effectively and extend food shelf life while regulating water exudation. Chitosan-based hydrogels prepared in the form of films have different food packaging and different physicochemical characterization compared to those in the form of gels. Tian B et al. (B. Tian, Wang, Liu, Liu, & Chen, 2021) prepared nano silica hydrogel films with attached β-acid for food packaging using chitosan as a substrate. The film showed improved properties such as mechanical strength, water vapor permeability, antimicrobial properties, and active substance release effect, thus becoming a promising material for food packaging. The improved resistance to ultraviolet light, antimicrobial effect, water absorption, and slow-release effect are very important factors in packaging food containing β-acids, which can better achieve the goal of long-term food storage.

### 4.3 As food dye adsorbents

Global attention has been increasingly drawn to the environmental challenges resulting from the contamination of food wastewater. One significant contributor to this issue is dye wastewater, which poses several inherent challenges due to its non-biodegradable nature as a macromolecule. This wastewater is characterized by high toxicity, rendering its removal process challenging. Additionally, it exhibits strong chemical resistance and delayed breakdown, which further complicates its treatment (Dimpee Sarmah & Karak, 2020). The persistence of dye wastewater in the environment can have severe direct consequences on various species and pose a threat to biological health. It is worth noting that the structural stability of dye wastewater adds to its resilience, particularly in photosynthetic water systems (Goncalves, da Silva, Rios, Crispim, Dotto, & de Almeida Pinto, 2020). The understanding and mitigation of these complexities are essential for addressing the global environmental impacts associated with food wastewater contamination.

**Fig. 3:**
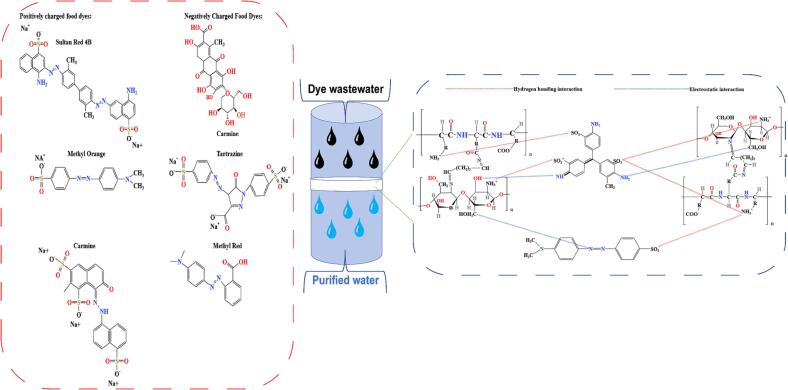
(a) Food dyes with different charges (b) Adsorption mechanism of food dyes in wastewater on CMCS/PVA hydrogels.

As a dye adsorbent, chitosan hydrogel is distinguished by its exceptionally low energy consumption, easy and quick operation, great flexibility, high degradability, and high adsorption rate (Lu et al., 2018, Liu et al., 2018). Because of its high swelling property, it may enhance the transit of water and dye, causing the binding sites in the hydrogel to bond with the dye, thus improving the adsorption effect. Because of the enormous amount of hydroxyl (–OH) and amino (–NH_2_) functional groups in chitosan molecules, it may physically adsorb food colors (de Farias et al., 2018). Chitosan has comparable properties to cationic polymers in acidic aqueous solutions; therefore, it may be successfully coupled with anionic contaminants in wastewater to boost the removal effectiveness of food diesel (Qiao, Ma, Wang, & Liu, 2021). Huy Q. Le et al (Le, Sekiguchi, Ardiyanta, & Shimoyama, 2018) synthesized chitosan hydrogels by activating chitosan with carbon dioxide (CO_2_) and then tested the adsorption and removal effectiveness of vivid blue FCF and Congo red food dyes at various temperatures. The combination of chitosan with CO_2_ resulted in the formation of three functional groups (protonated amino group, carbamate, and bicarbonate), the NH_2_ group of which may be employed for dye adsorption sites and cross-linking. Carbon dioxide is activated in the chitosan network and can create carbamate cross-links. The activation energy of the carbohydrates produced by amine and carbon dioxide (CO_2_) lowers at high temperatures, increasing the stability of chitosan in carbon dioxide (CO_2_) treatment systems. Since carbon dioxide solubility or aqueous pH has no effect on the stability of chitosan at increased temperatures, the carbon dioxide-activated hydrogel becomes increasingly effective for dye removal as the temperature rises. Huy Q. Le et al. (Zhang et al., 2023, Lv et al., 2023, Zhang et al., 2023) successfully prepared a dual network hydrogel (CMCS/PVA) using carboxymethyl chitosan (CMCS) and poly (vinyl alcohol) (PVA) as materials by freeze-thawing and cross-linking with calcium chloride phase. –OH, in PVA and –COOH in CMCS were physically crosslinked throughout the preparation process through hydrogen bonding interactions, resulting in the formation of a hydrogel with a three-dimensional network structure. A tighter link formed between CMCS and PVA because of the simultaneous esterification of the PVA ester bond with CMCS. The carboxyl anion in carboxymethyl chitosan then interacted with the positively charged calcium ions in calcium chloride because of the interaction with the additional calcium chloride, leading to the creation of a hydrogel with a dual network structure. The increased pore structure of the hydrogels produced from the preparation provided a higher surface area for adsorption. The hydrogel's functional groups, such as –NH_2_, –COOH, and –OH, served as adsorption sites that improved the hydrogel's ability to bind to AB dyes through the interactions of electrostatic force, hydrogen bonding, and van der Waals' force (Fig. 3).

### 4.4 Delivery of nutrients as encapsulated material

Chitosan hydrogel beads are spherical particles in a hydrogel matrix that take advantage of the hydrogel's outstanding qualities to ensure the protection and controlled release of core chemicals. To satisfy the demands of diverse areas, the size, shape, and active ingredient release properties of the gel beads may normally be modified by changing the cross-linking conditions (Karzar Jeddi & Mahkam, 2019). In functional foods and nutritional supplements, for example, regulating the release of nutrients to give the body the necessary nutritional supplements to obtain an appropriate release rate and a long-lasting impact is utilized (Liu et al., 2023, Liu et al., 2023). Huang J et al. (J. Huang, Wang, Chu, & Xia, 2020) used ionic cross-linking injection gelation to create a liposome-chitosan hydrogel bead with linseed oil and quercetin as the core. The resultant hydrogel beads had a three-dimensional network structure that was designed to slow the release rate of bioactive, hence increasing their absorption and utilization efficacy in organisms. Following characterization, the produced hydrogel beads demonstrated a smooth surface and solid internal structure, as well as good slow-release performance and stability in simulated intestinal digesting fluids. The hydrogel beads significantly reduced the oxidative reaction and UV radiation destruction of the bioactive, increasing the bioavailability of the orally given medication for greater absorption of quercetin by the human body. In addition to studying the absorption and stability of gel beads in the human body, investigations on their nutrition release capabilities are required. Duffy C et al. (Duffy, O'Sullivan, & Jacquier, 2018) used the encapsulated iron approach and extrusion method to create a new chitosan hydrogel bead. The results revealed that chitosan hydrogel beads produced with ammonium ferric citrate (III) (AFC) had improved swelling and release properties in water and milk, as well as a high iron ion retention capacity. The gel beads had a good release rate (without impacting the pH change), and the amount of iron released was sufficient to fulfill the necessary consumption for adults, according to simulated gastric fluid in vitro release trials. With the addition of iron, lipid oxidation was decreased, and the resulting rancidity perception threshold was close to the rancidity threshold reported in food sensory testing.

## 5 Conclusion and prospect

With growing interest in hydrogel substrates, the selection of safe and efficient materials for hydrogel fabrication has become critical to promoting their growth in the food area. Chitosan is an ideal biopolymer with a unique structure and good biocompatibility, biodegradability, anti-bacterial and antioxidant activities, which has piqued the interest of many researchers and is currently a significant material in the study and development of hydrogels. Chitosan hydrogels are now employed mostly in the sectors of medical devices, biomaterials, and drug discovery, and are not frequently used in the food industry.

As the food industry has evolved, chitosan-based hydrogels have been used as food packaging materials, food color adsorbents, and food sensors. However, the use of chitosan-based hydrogels in food is still in its early stages. For researchers, there are still several difficulties to overcome. For example, (1) chitosan-based hydrogels may introduce toxic chemicals during the manufacturing process, and there may be safety hazards when used in food; (2) the application of chitosan hydrogels in food, such as edible packaging materials and food conditioners, requires additional investigation. (3) chitosan's low water solubility makes direct application difficult, necessitating the creation of chitosan derivatives. However, the use of potentially hazardous chemicals in the synthesis of these derivatives might constitute a safety risk. (4) At the moment, several issues remain in the research of the formation process and mechanism of action of chitosan-based hydrogels, such as the change in polymer chemical bonding during gelation of chitosan-based hydrogels, which requires further in-depth theoretical and statistical support.

This review examined the various cross-linking techniques and biological activities of chitosan-based hydrogels, as well as recent applications of chitosan hydrogels in the food industry, to overcome the challenges of typical hydrogels' poor mechanical strength, short preservation period, and limited application scope. Exploring the application of chitosan hydrogels in the food field, as well as the differences and connections between their preparation mechanisms and cross-linking methods is a future research direction that will serve as a reference for future hydrogel development and utilization, as well as promote their further development in the food field.

## Informed consent

Not applicable.

Ethical guidelines

Ethics approval was not required for this research.

## CRediT authorship contribution statement

**Fandi Hong:** Writing – original draft. **Peng Qiu:** Data curation. **Yufan Wang:** Methodology. **Peirou Ren:** Methodology. **Jiaxin Liu:** Supervision. **Jun Zhao:** Resources. **Dongxia Gou:** Conceptualization, Writing – review & editing.

## Declaration of competing interest

The authors declare that they have no known competing financial interests or personal relationships that could have appeared to influence the work reported in this paper.

## Data Availability

No data was used for the research described in the article.
